# Calcium electroporation of esophageal cancer induces gene expression changes: a sub-study of a phase I clinical trial

**DOI:** 10.1007/s00432-023-05357-y

**Published:** 2023-09-09

**Authors:** Charlotte Egeland, Lukas Balsevicius, Ismail Gögenur, Julie Gehl, Lene Baeksgaard, Rajendra Singh Garbyal, Michael Patrick Achiam

**Affiliations:** 1 Department of Surgery and Transplantation, Copenhagen University Hospital Rigshospitalet, Copenhagen, Denmark; 2 Department of Clinical Medicine, Faculty of Health and Medical Sciences, University of Copenhagen, Copenhagen, Denmark; 3 Center for Surgical Science, Department of Surgery, Zealand University Hospital, Koege, Denmark; 4 Graduate School of Health and Medical Sciences, University of Copenhagen, Copenhagen, Denmark; 5 Department of Clinical Oncology and Palliative Care, Center for Experimental Drug and Gene Electrotransfer (C*EDGE), Zealand University Hospital, Roskilde, Denmark; 6 Department of Oncology, Copenhagen University Hospital Rigshospitalet, Copenhagen, Denmark; 7 Department of Pathology, Copenhagen University Hospital Rigshospitalet, Copenhagen, Denmark

**Keywords:** Esophageal cancer, Calcium electroporation, Gene expression changes, Cellular composition, Immunological response, Tumor microenvironment

## Abstract

**Purpose:**

In this study, we aim to investigate gene expression changes in tumor samples obtained from patients with esophageal cancer treated with calcium electroporation. Previously, local treatment with calcium electroporation has been shown to induce gene expression alterations, potentially contributing to a more tumor-hostile microenvironment.

**Methods:**

In this sub-study of a phase I clinical trial, we included five patients with esophageal cancer treated with calcium electroporation. We compared cancer-associated gene expression patterns in tumor samples before and after treatment. Furthermore, we used linear support vector regression to predict the cellular composition of tumor samples.

**Results:**

Using differential expression analysis, we identified the downregulation of *CXCL14* and upregulation of *CCL21*, *ANGPTL4*, and *CRABP2* genes. We also found a decreased predicted proportion of dendritic cells while the proportion of neutrophils was increased.

**Conclusion:**

This study provides evidence that calcium electroporation for esophageal cancer induces local transcriptional changes and possibly alters the cellular composition of the tumor microenvironment. The results are explorative, larger studies are needed to confirm and further correlate our findings with clinical outcomes.

**Supplementary Information:**

The online version contains supplementary material available at 10.1007/s00432-023-05357-y.

## Introduction

Esophageal cancer (EC) is one of the most lethal cancer types, with an overall 5-year survival of only 20% (American Cancer Society 2022). Close to 40% of the patients have metastatic disease already when diagnosed (5-year survival: 6%) (National Cancer Institute 2020) and are only candidates for palliative treatment. Palliative care consists of chemotherapy, radiotherapy, and local treatments are important in relieving local symptoms. Reversible electroporation facilitates the transport of ions and molecules across the cell membrane by short electrical pulses. The pulses permeabilize the cell membrane and temporarily allow otherwise impermeable (or poorly permeable) molecules to enter the cytosol. This technique is used in electrochemotherapy (ECT), in combination with a cytostatic drug (typically bleomycin), and in calcium electroporation (CaEP), a procedure involving the injection of calcium into malignant tissue, enabling it to subsequently penetrate permeabilized tumor cells and trigger cell death. Our group has previously investigated endoscopic-assisted ECT (Egeland et al. 2018) and CaEP Egeland et al. (2022) as local supplemental treatment in patients with non-curable EC.

A sufficient immune response is necessary to combat cancer by enabling the detection, elimination, and prevention of cancer cells and providing immunological memory and adaptability. Enhancing immune responses against cancer has been a significant focus in cancer research. It has led to the development of immunotherapies that harness and strengthen the immune system's ability to fight cancer (Hanahan and Weinberg 2011). Immune checkpoint inhibitors that target programmed cell death protein 1 (PD-1) receptors, such as pembrolizumab and nivolumab, have been approved by regulatory authorities for the treatment of advanced EC (Doki et al. 2022; Sun et al. 2021). Novel strategies use a complementary combination of two different therapy forms, for example, irradiation or chemotherapeutic drugs and an immunotherapeutic drug. Here, the direct local cytotoxic effect, which causes immunogenic cell death, and the stimulation or inhibition of immune checkpoints create an enhanced effect compared with only applying the treatments separately (Arina et al. 2020; Galluzzi et al. 2020).

From preclinical studies, both ECT and CaEP have been shown to give rise to an anticancer immune response and to repress distant tumor growth outside of the treated area (Calvet et al. 2014; Di Gennaro et al. 2016; Falk 2008, 2017; Tremble et al. 2019). ECT is known to induce immunogenic cell death by releasing High Mobility Group Box 1 Protein (HMGB1), calreticulin, and ATP (Calvet et al. 2014). In murine models, ECT has been shown to induce a systemic effect and suppress distant metastases (Roux et al. 2008; Tremble et al. 2019). In colon cancer mouse models, CaEP and ECT created a systemic, long-lasting protective immunity by increasing the release of HMGB1 and the overall systemic level of pro-inflammatory cytokines (Falk 2017) and recently, it was shown that the effect of CaEP was further potentiated by Interleukin-12 in tumor-bearing mice (Lisec et al. 2023).

Despite these preclinical findings, there is still a lack of mechanistic understanding and what immunological effects can be expected in clinical trials. However, in one case report, a patient with disseminated malignant melanoma experienced a systemic response after only local treatment with ECT and CaEP (Falk 2017). This has led to the hypothesis that both the local and the systemic immune response can be further enhanced by combing ECT or CaEP with immunotherapeutic drugs. Several smaller studies have investigated ECT in combination with immunotherapy in patients with skin cancer with promising results (Andersen et al. 2003; Campana et al. 2021; Heppt et al. 2016; Hribernik et al. 2016; Mozzillo et al. 2015; Theurich et al. 2016). Even though CaEP as a monotherapy has been investigated in several cancer types (Ágoston et al. 2020; Broholm et al. 2023; Egeland et al. 2022; Falk et al. 2018; Jensen et al. 2022; Plaschke et al. 2019; Stranzenbach et al. 2021; Yousra et al. 2021), CaEP in combination with immunotherapy has only been reported casuistically. In a case of disseminated urothelial cancer, long-term management of regional recurring metastases was accomplished by employing a combination of systemic immunotherapy and local treatment utilizing CaEP, despite prior treatment failure with pembrolizumab as monotherapy (Vissing et al. 2023).

We hypothesized that local treatment with CaEP in patients with EC could lead to favorable changes in the tumor microenvironment, which could be associated with systemic anti-tumor effects.

## Materials and methods

### Study design

This is a sub-study of a phase I clinical study which has been published previously (Egeland et al. 2022). The study was approved by the Danish Medicines Agency (EudraCT no.: 2020-005787-58), the Regional Ethics Committee (H-20082119), and the Regional Department of Research and Innovation. The Good Clinical Practice Unit at Copenhagen University Hospital monitored the trial. We conducted the trial in accordance with the declaration of Helsinki (World Medical Association 2013), and written consent was obtained from all participants.

The full method from the clinical trial, including inclusion and exclusion criteria, is described in the published paper (Egeland et al. 2022). Briefly, eight patients with non-curable EC (57–83 years, six males and two females), and no other oncological treatment options, were treated with endoscopic-assisted CaEP. The electrical pulses were applied to the tumor area via an electrode attached to the endoscope, and Calcium gluconate (0.23 mmol/l) was injected intratumorally. Patients with both adenocarcinomas and squamous cell carcinomas were included. All patients had disseminated disease. After treatment, a visual tumor response was seen in five of the seven patients evaluated with an upper endoscopy. From the CT evaluation, one patient had a partial tumor response, and three patients had stable disease. No visual response was seen outside of the treated area.

### Sample collection and preparation

If the patient consented, biopsies were taken endoscopically from the tumor site before CaEP and again within seven days after treatment. The biopsies were immediately put in tubes containing *RNAlater*™ Stabilization Solution (Thermo Fisher Scientific, Waltham, MA, United States) and hereafter stored at − 80 °C. An experienced gastrointestinal pathologist assessed all biopsies. By microdissection of formalin-fixed paraffin-embedded (FFPE) blocks, non-malignant mucosa was excluded, and the thin blocks were stained with hematoxylin and eosin. Thus, only biopsies containing > 50% malignant tissue were selected for analysis. Total RNA was extracted from the samples using the High Pure FFPET RNA Isolation Kit (Roche Life Science, Penzberg, Germany). Approximately 300 ng of total RNA was obtained from each sample for gene expression quantification. The RNA was quantified using spectrophotometry (NanoDrop, Thermo Fisher Scientific, Waltham, MA, United States), and a quality assessment was performed (Bioanalyzer, Agilent, Glostrup, Denmark). Overnight RNA hybridization was conducted using the nCounter® IO360 panel for the 750 endogenous human transcripts (NanoString, Seattle, WA, United States). The complete gene panel is available from the manufacturer’s website (Nanostring PanCancer Human IO360 Panel Gene List 2023).

### Gene expression analysis

Raw data from the nCounter® platform was pre-processed through an iterative quality control and normalization framework (Bhattacharya et al. 2021). To evaluate technical sample quality, principal component analysis (PCA) plots, relative log expression (RLE) plots, and Spearman correlation heatmaps were developed. Raw gene counts were normalized by first running upper quartile normalization, followed by variance stabilizing transformation. Unwanted variation was estimated using the “RUVg” function from the RUVSeq package (v.1.32.0). Samples not passing the iterative quality control were excluded from further analysis (outliers with their corresponding paired samples). After discarding poor-quality samples (*n = *4), we removed no (*n = *0) vectors of unwanted variation.

To identify differentially expressed genes (DEGs) before and after CaEP treatment, we used Wald significance test within the DESeq2 analysis framework (“DESeq” function from DESeq2 package (v.1.38.0)) (Love et al. 2014). Differential expression (DE) experiment design formula was set as =  ~ *Patient_ID* + *Time_point*. The threshold requirement for significant DEGs was |log2FC|≥ 1 and an adjusted *p* value ≤ 0.05 (Benjamini–Hochberg correction). Log2FC value > 1 indicated a higher gene expression after CaEP treatment, while a log2FC value < − 1 indicated a lower gene expression after treatment. The “EnhancedVolcano” package (v.1.11.1) and ggplot (v.3.4.0) were used to visualize the DEGs (Blighhe et al. 2021).

We used the top (*n = *100) most variable features for PCA to minimize technical noise and keep only the most informative features (genes). PCA was performed, and results are presented using the “pca” and “biplot” functions from *PCAtools* package (v.2.5.3) (Blighe et al. 2022). To determine the features that drive most of the variation in PC space, we extracted the most variable features from each PC that fall within the top/bottom (*n = *20) of each PC loading.

Linear support vector regression [31] was applied for cell type deconvolution of the gene expression data. The “safeTME” dataset (Danaher et al. 2022) was filtered and adjusted to include signatures for T cells (CD4 + and CD8 +), B/Plasma cells, monocytes/macrophages, neutrophils, mast cells, dendritic cells, and non-immune cells. The algorithm was run with 1000 permutations. The “ggplot2” package (v.3.4.0) was used to visualize the predicted proportions of cell types.

### Statistical analysis

R was used for all statistical analyses (R Development Core Team 2010). To evaluate if numerical variables in the gene expression panel were distributed normally, we generated distribution histograms and performed a Shapiro–Wilk normality test. Bar plots were illustrated as the median with interquartile ranges (IQR). Boxplots showed as the median with the lower and upper hinges corresponding to the first and third quartiles (the 25th and 75th percentiles) and whiskers extending from the hinge to the largest/lowest value no further than 1.5 * IQR from the hinge. Data input for PCA and cell type deconvolution analyses were normalized gene counts, while raw counts were used in the DEG analysis. Permutational multivariance analysis of variance using distance matrices (PERMANOVA) was used to qualitatively assess group differences in the PCA. All statistical comparisons between group differences were made using the Wilcoxon rank-sum test unless stated differently. Adjusted and un-adjusted *p* values ≤ 0.05 were considered statistically significant.

## Results

### Sample selection and patient characteristics

**Fig. 1:**
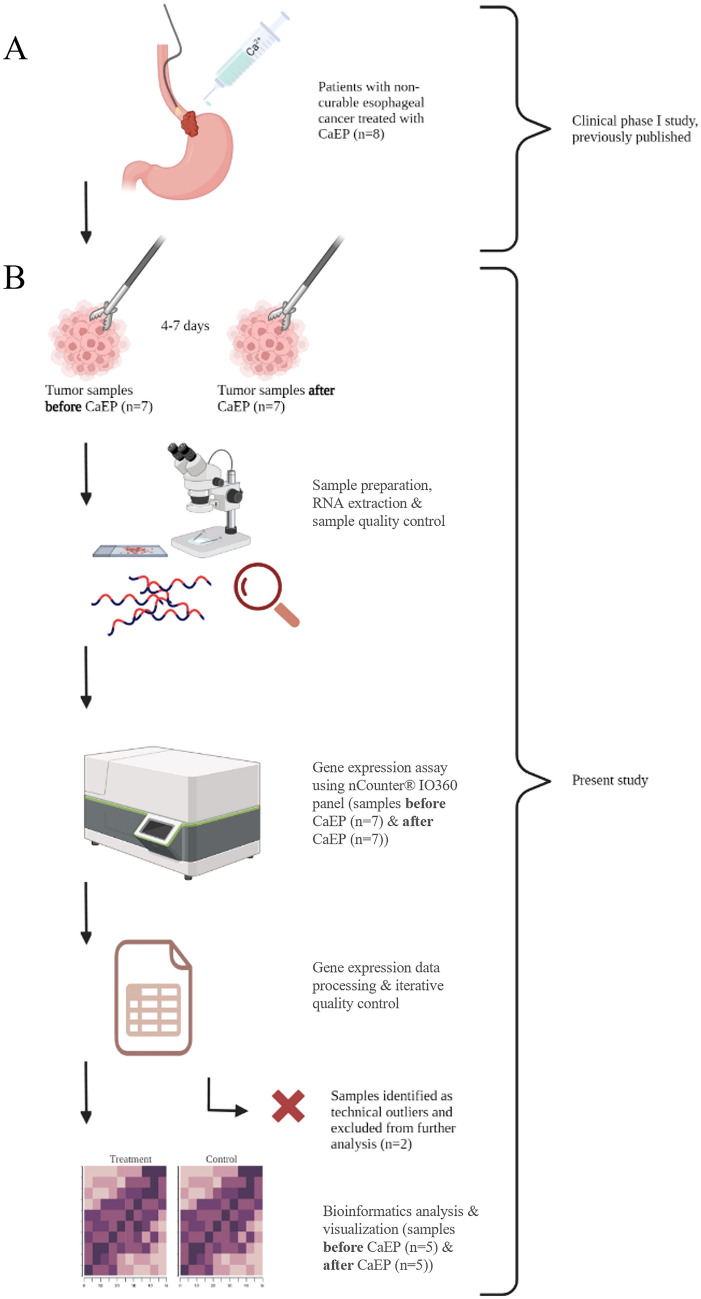
Study overview. **A** Clinical phase I study where eight patients with non-curable esophageal cancer were treated with endoscopic-assisted CaEP (Egeland et al. 2022). Reversible electroporation was applied to the tumor area, followed by intratumoral injection of Calcium gluconate (0.23 mmol/l). **B** Biopsies were taken from the tumor before and after treatment from seven patients. The samples were assessed by a pathologist, RNA was extracted, and after iterative qualitative control, ten samples were included in the final gene expression analysis. CaE*P = *Calcium electroporation. This image was created with Biorender.com

In the clinical study (Egeland et al. 2022), we collected biopsies from seven of the eight treated patients (2021–2022). During patient selection for this sub-study, by iterative quality control, two post-treatment samples were identified as potential technical outliers (“ID_2_PostT” & “ID_5_PostT”). To preserve the experimental setup, in which we investigate paired sample temporal changes, we also removed baseline samples of these two patients (“ID_2_Baseline” & “ID_5_Baseline”). A study overview, including sample selection after quality control, is presented in Fig. 1. The iterative quality control process, including raw and normalized data (after excluding patient ID_2 and ID_5), is shown in Supplementary Figures S1 and S2. Cumulatively, paired samples from five patients (number of samples = 10) were included in the downstream analyses. Baseline characteristics and treatment data for the included patients are presented in Table 1.

**Table 1:** Characteristics

Patient ID	Gender/Age	Tumor Type	Disseminated disease	Treatment Data	PostT biopsy obtained
1	Male/76 yr	Adc	Yes	20 mL (0.23 mmol/l) calcium gluconate 7 pulses	POD 7
3	Male/59 yr	Adc	Yes	20 mL 7 pulses	POD 6
4	Male/62 yr	Adc	Yes	19 mL 12 pulses	POD 6
6	Female/66 yr	Adc	Yes	19 mL 20 pulses	POD 4
7	Female/72 yr	Adc	Yes	20 mL n/a pulses	POD 7

Baseline characteristics and treatment data for the five included patients in the final gene expression analysis

*Adc* adenocarcinoma; *PostT biopsy* post-treatment biopsy; *POD* postoperative day

### Calcium electroporation induces alterations in gene expression patterns

**Fig. 2:**
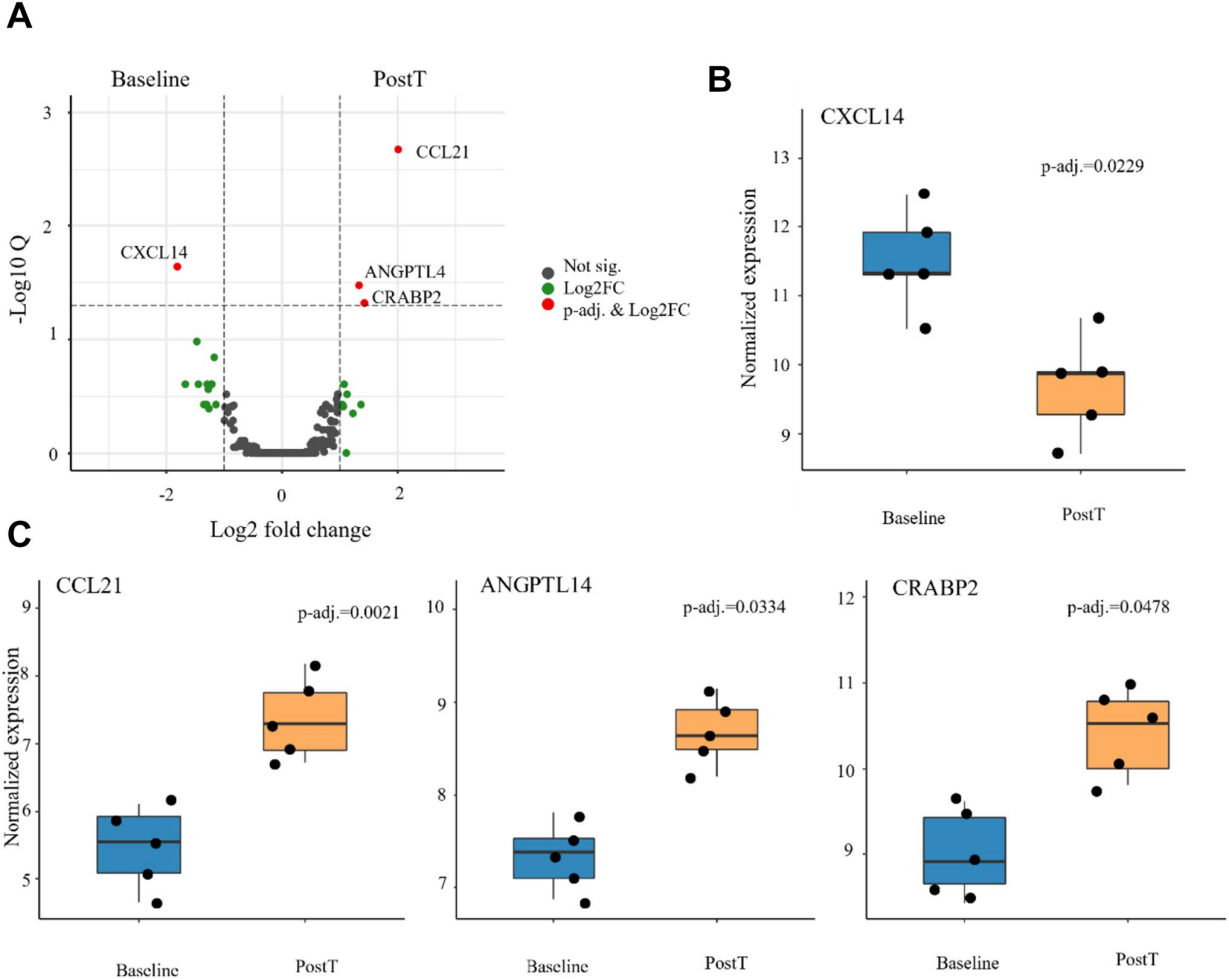
Calcium electroporation induces changes in gene expression. **A** Volcano plot illustrating differentially expressed genes (DEGs). |Log2FC|≥ 1 and an adjusted *p* value ≤ 0.05 (Benjamini–Hochberg correction) were required for significance. Log2FC > 1 indicated an upregulated gene expression, while log2FC < − 1 indicated downregulated gene expression after CaEP. **B**, **C** DEGs illustrated as boxplot with median, upper and lower quartiles, whiskers extend into a max of 1.5 times the IQR, representing the four individual genes statistically differentially expressed after treatment. *CXCL14* was downregulated (**B**), and *CCL21, ANGPTL4*, and *CRABP2* were upregulated (**C**) after treatment. PostT—Post treatment

Four genes were identified as statistically differentially expressed when comparing samples before and after CaEP treatment (Fig. 2A). *CXCL14* (Chemokine ligand 14) was found to be downregulated (Fig. 2B, Log2FC < − 1, adjusted *p = *0.023). *CXCL14* is associated with the attraction and maturation of immune cells and the motility of epithelial cells. *CCL21* (Chemokine ligand 21), *ANGPTL4* (Angiopoietin-like 4), and *CRABP2* (Cellular retinoic acid-binding protein 2) were found to be upregulated (Fig. 2C, Log2FC > 1, adj. *p = *0.002, adj. *p = *0.003, and adj. *p = *0.048, respectively), after treatment. These genes play a role in the attraction of antigen-presenting dendritic cells and T-cells (*CCL21*) and regulation of proliferation, apoptosis, and invasion (*ANGPTL4* and *CRABP2*).

**Fig. 3:**
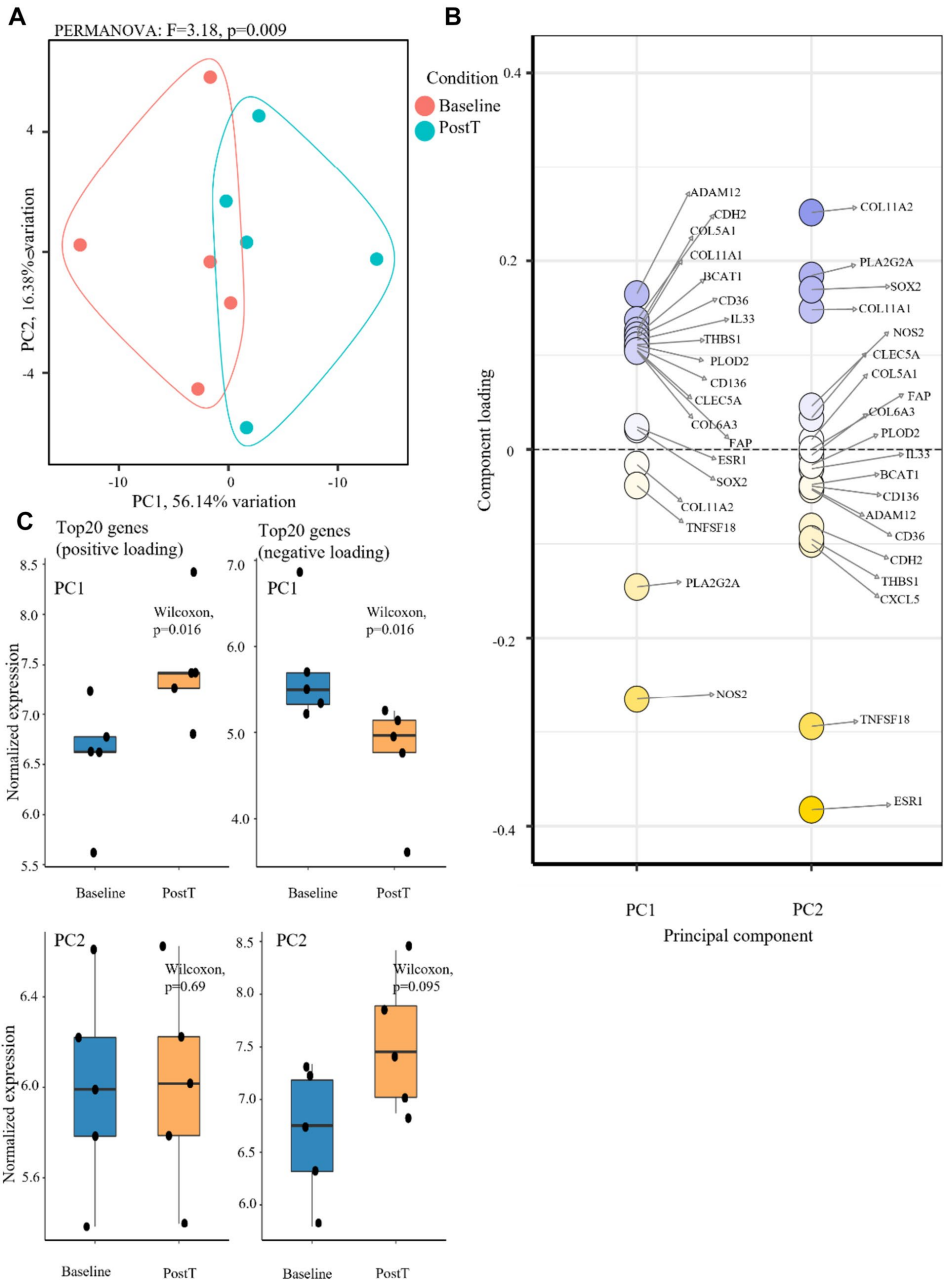
Calcium electroporation affects tumor gene expression. **A** Principal component (PC) analysis based on the top 100 most variable genes from nCounter® IO360 panel. PERMANOVA was used to test for statistical significance between time points. **B** Top 20 genes contributed to PC loading in PC1 and PC2, respectively. Component loading > 0 indicates positive and component loading < 0 indicates negative coefficients of top variables (genes) from which PCs are constructed. **C** Boxplots illustrating up- and downregulated components in PC1 (top) and PC2 (bottom), respectively. Wilcoxon rank-sum test was used to test for statistical significance. *PostT* post treatment

Figure 3 shows the results from the PCA. Principal components PC1 and PC2 contributed to 56.1% and 16.4% of the total variance, respectively. The PCA shows clear separation by PC1 between pre-treatment and post-treatment samples (Fig. 3A, paired PERMANOVA: *F = *3.18, *p = *0.009). The specific genes that are the main features responsible for separation in principal component space are shown in Fig. 3B. Figure 3C illustrates the difference in both negative and positive loading in PC1 and PC2, respectively. Genes encoding collagen components (*COL5A1* and *COL11A1*), *ADAM12* which is associated with stromal factors, and *CDH2* encoding cadherin-2 protein (associated with malignant cell migration) are the main positive features of PC1 (upregulated). *NOS2*, a gene associated with inflammation, infection control, and immune regulation, was found to be the top negative feature of PC1 (downregulated). On the other hand, genes included in PC2 were not statistically significantly responsible for sample separation. Genes in PC2 include, among others, *ESR1* (known to be a tumor suppressor gene) and *TNFSF18* (associated with the modulation of T lymphocytes) (top negative features), and *COL11A2*, encoding collagen components (top positive feature).

### Calcium electroporation affects the predicted cellular composition of the tumor microenvironment

**Fig. 4:**
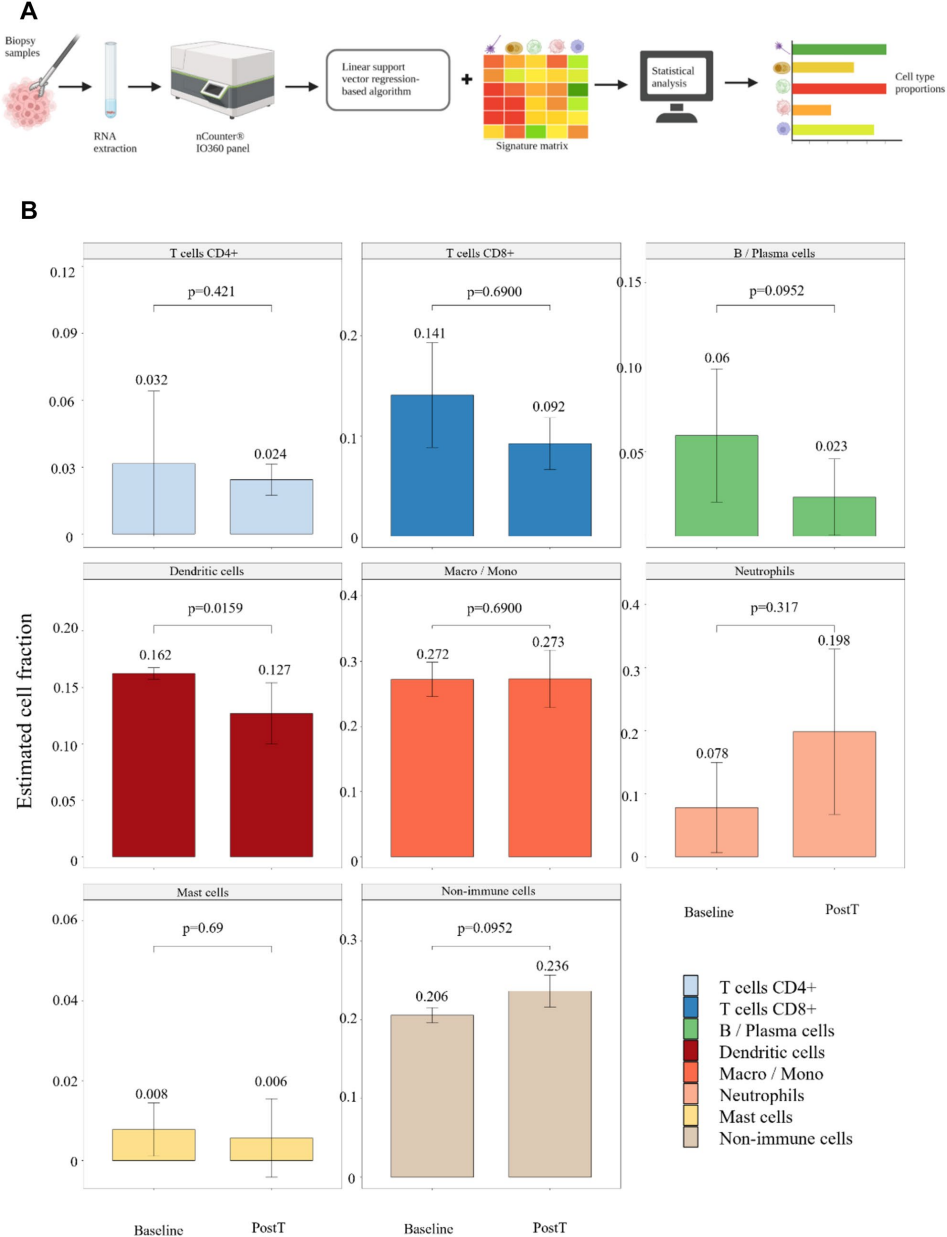
Calcium electroporation alters predicted cell type proportions. **A** Infographic depicting the cell type prediction experiment design. **B** Boxplots with median and IQR of the included cell types. The predicted proportion of dendritic cells was reduced, and the predicted proportion of neutrophils was elevated after CaEP. *PostT* post treatment. **A** is created with Biorender.com

We used linear vector regression to predict cell type abundances consisting of eight different cell types (CIBERSORTx workflow) from the gene expression data, Fig. 4A. We ran the comparison of the predicted cell type proportions before and after treatment, Fig. 4B. Two of the inferred cell type fractions were found to be statistically significantly different. The fraction of dendritic cells was reduced (*p = *0.016), and the fraction of neutrophils was elevated (*p = *0.032), suggesting a reprogramming of the innate immune cell compartment. No changes were found in the fractions of predicted T cells (CD4 + and CD8 +) or B/Plasma cells, indicating a lack of effect on adaptive immunity. Non-immune cell fraction consisting of malignant and non-malignant epithelium was also not affected by the CaEP treatment.

## Discussion

This is the first study investigating changes in primary tumor gene expression patterns in esophageal cancer after treatment with CaEP. Within seven days after treatment, we found significant differences in gene expression, and the predicted abundance of both dendritic cells and neutrophils was altered.

Using differential gene expression analysis, we found that *CXCL14* was downregulated after treatment. *CXCL14* is normally expressed in high levels in benign cells, but the expression is mostly reduced or completely absent in malignant cells (Hromas et al. 1999). The role of *CXCL14* is known to be associated with the attraction and maturation of immune cells and the local infiltration of dendritic cells (Shurin et al. 2005). Dendritic cells are antigen-presenting cells that activate T-cells, and an increase in dendritic cells is often described as associated with better clinical outcomes (Huang and Fu 2019). In this study, we found that the fraction of dendritic cells decreased after CaEP, possibly contributing to the shown downregulation of the *CXCL14* gene. Dendritic cells are believed to be crucial for the effect of PD-1 inhibitors (Garris et al. 2018), which is an immunotherapeutic drug approved for the treatment of advanced esophageal cancer. In theory, the reduction in tumor-infiltrating dendritic cells could lead to an impaired effect of PD-1 inhibitors in combination with CaEP. Across studies, the results are inconsistent regarding the association between *CXCL14* expression and cancer survival, and further, this has not been investigated in EC specifically. A high expression of CXCL14 is associated with better overall survival in colorectal, cervix, head and neck, endometrial, and breast cancer. At the same time, other studies have found that the expression levels positively correlate with a more aggressive type of prostate cancer and poorer patient survival in malignant melanoma (Westrich et al. 2020). The conflicting results indicate that the role of CXCL14 is highly context-dependent, and that the protein interacts with various other factors in the tumor microenvironment. Hence, the clinical meaning of the downregulation of *CXCL14* and reduced fraction of dendritic cells after CaEP awaits further elucidation, even though current scientific knowledge links such changes in the tumor microenvironment with unfavorable outcomes.

*CCL21, ANGPTL4*, and *CRABP2* were upregulated after the treatment. CCL21 acts as a ligand to the CC-chemokine receptor 7 (CCR7). When the CCR7 receptor is activated, it helps to localize and attract antigen-presenting dendritic cells and T-cells (Alrumaihi 2022). As mentioned above, we found a lower expression level of dendritic cells, while no changes were observed in the expression level of T-cells. In human colorectal cancer tissue samples, a higher *CCR7* receptor expression level was associated with poorer overall survival (Nagasawa et al. 2021). *ANGPTL4* gene encodes Angiopoietin-like 4 protein (ANGPTL4) that is a part of the ANGPTL protein family, and these proteins are known to play a role in carcinogenesis and metastases development, especially by modulating the angiogenesis (Carbone et al. 2018). Most evidence points toward ANGPTL4 primarily acting as a tumor promoter, disrupting vascular tight junctions and increasing the capillary permeability (Westrich et al. 2020), and facilitating vascularizing on which malignant cells are highly dependent. However, other studies suggest that the function of ANGPTL4 is highly tumor-type dependent, and the protein might act as an anti-angiogenic protein in some cancer types as its role may be altered depending on the proteolytic cleavage and posttranslational changes (Carbone et al. 2018). ANGPTL4 further seems to inhibit other cell death promotors, helping the malignant cells avoid apoptosis (Tan et al. 2012). Two clinical studies have investigated ANGPTL4's specific prognostic role in esophageal cancer, including esophageal squamous cell carcinoma. Both concluded ANGPTL4 to be associated with more aggressive disease (Shibata et al. 2010; Yi et al. 2013). Similar results have been published in preclinical trials in gastric cancer (Chen et al. 2018). Therefore, the upregulation of *CCL21* and *ANGPTL4* in this trial might favor more aggressive disease and not necessarily benefit tumor suppression. Lastly, *CRABP2* encodes for a protein (CRABP2) associated with the regulation of proliferation, apoptosis, invasion, and metastasis. It is described as both an oncogene and a tumor suppressor gene. A single study suggested CRABP2 to acts as a tumor inhibitor in esophageal carcinoma (Yang et al. 2016), while in breast cancer, CRABP2 can both suppress or promote tumor invasion depending on tumor type (Feng et al. 2019). Analyses from esophageal tissue samples showed that the expression of CRABP2 was lower in malignant tissue than in healthy epithelial tissue. No survival difference was found between patients with high versus low expression of CRABP2 (Li et al. 2021). These findings suggest that the upregulation of the *CRAPBP2* gene could have a positive, tumor-suppressing effect.

From the PCA, genes encoding collagen components were upregulated after treatment. Collagen is one of the significant components in the tumor microenvironment as it provides structural support to the extracellular space of connective tissue and is associated with cancer cell invasion, proliferation, and metastases, regulation of intratumoral vessels, and cancer cell death resistance (Xu et al. 2019). One study demonstrated that a high expression of COL11A1 in patients with EC was associated with poorer overall survival (Zhang et al. 2018). However, it is unknown whether an increase in gene expression before and after a specific treatment (seen in this study) has the same impact as an absolute high expression compared with healthy tissue. Furthermore, *ADAM12* was part of the top positive features of PC1. In human gastric cancer cell lines, *ADAM12* enhanced tumor cell migration and invasion and inhibited apoptosis, which was further correlated with poorer survival (Chung et al. 2022). *NOS2* was the top negative feature of PC1 and is encoding for the protein Nitric Oxide Synthase. NOS2 has been demonstrated to possess anti-tumoricidal functions and to predict poor patient outcomes in several cancer types, including stomach and colon cancer, as it correlates with increased vascularization and metastasis (Thomas and Wink 2017). Therefore, it has been suggested as a promising target for cancer therapy. A decrease in the expression of *NOS2* could indicate a more tumor-hostile environment and a potential clinical benefit.

A recently published trial examined the tumor microenvironment by immunofluorescence staining of immune cells in tumor-bearing mice after treatment with CaEP and Interleukin-12 (Lisec et al. 2023). The authors found a significant increase in infiltration of Natural Killer (NK) cells and CD8 + T-cells in both tumor models. In one tumor model, there was a significant decline in Ki-67 + cells. The Ki-67 + protein is a cellular market for cell proliferation. The gene *MKI67* (encodes Ki-67 protein) was part of the gene panel in this trial but was not statistically altered after treatment. We observed no difference in CD8 + T-cell levels, while NK-cells were not part of our final model. On the other hand, we found that the fraction of neutrophils after treatment increased significantly. Most data during the last decade have indicated that neutrophils support cancer growth, while new evidence suggests that their role in cancer is dual, where they also exert anticancer effects (Xiong et al. 2021).

The current study has limitations. First, the sample size is small, with only five paired samples. Because biopsy in patients with esophageal cancer requires endoscopy, the number of samples will naturally be restricted, and there may also be considerations regarding time from the first to second biopsy procedure, taking consideration for the patient into account. The post-treatment samples were all obtained within a short time frame (4–7 days). In the pioneer study in mice (Falk et al. 2017a; b), gene expression changes were revealed in samples taken three days after treatment. When performing gene expression analyses with the nCounter® IO360 panel, mRNA expression is measured, not synthesized proteins' level. Due to different posttranslational alterations, mRNA expression does not necessarily correlate with protein expression. Furthermore, the function of a protein is highly dependent on its binding to specific ligands, which were neither examined in this trial. Several protein assays exist to quantify the level of functional proteins, which we main to integrate in our future follow-up studies together with mRNA expression measures to create a more holistic view of the molecular microenvironment. Furthermore, we have only examined 750 genes out of the full protein coding transcriptome, which could have led to unnoticed changes. Lastly, within the cell type prediction model, due to the limited amount of cellular markers in the targeted gene expression assay, we could not accurately infer some of the relevant immune cell types, e.g., NK cells, which would have provided valuable insights and their role will be assessed in the subsequent experiments.

## Conclusions

The present research provides additional evidence confirming the preclinical findings indicating that CaEP induces cellular and molecular alterations in the tumor microenvironment. *CCL21, ANGPTL4*, and *CRABP2* were upregulated, while *CXCL14* was downregulated after the treatment. Furthermore, CaEP led to a reduction in the fraction of dendritic cells and an increase in the fraction of neutrophils. To fully comprehend and assess the clinical significance, larger studies encompassing outcome and survival data are necessary.

## Supplementary Information

Below is the link to the electronic supplementary material.

Supplementary file1 (DOCX 293 KB)

## Data Availability

Raw and derived data supporting the findings of this study are available from the corresponding author (Charlotte Egeland) on request.
