# Supplementary material for: Calcium electroporation of esophageal cancer induces gene expression changes: a sub-study of a phase I clinical trial
Source: J Cancer Res Clin Oncol. 2023 Sep 9;149(17):16031–42. doi: 10.1007/s00432-023-05357-y (PMC10620256; doi:10.1007/s00432-023-05357-y)
Supplement: Supplementary file 1 — Supplementary file1 (DOCX 293 KB) [file 432_2023_5357_MOESM1_ESM.docx]

Supplementary **Fig S1** – Technical quality control


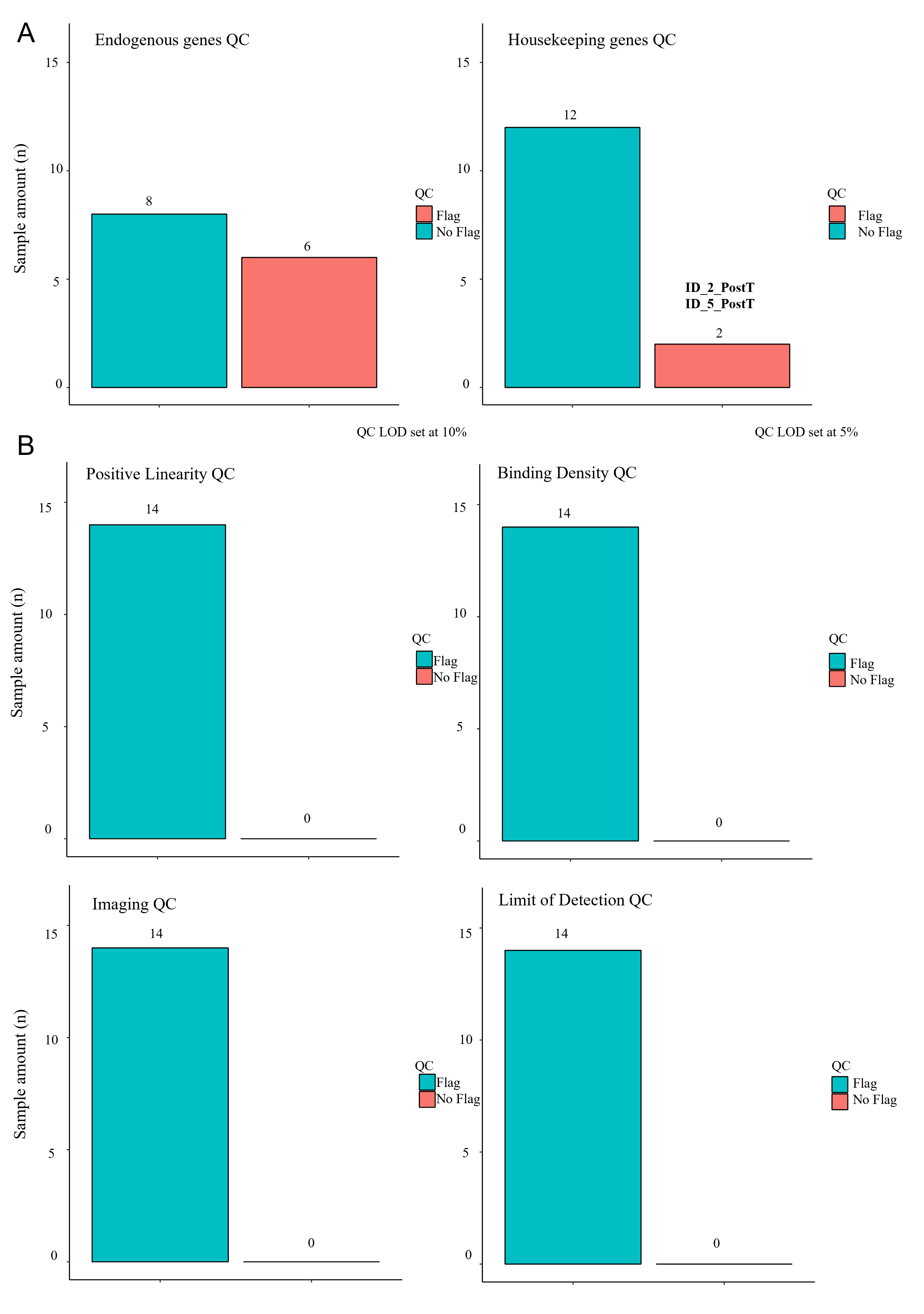


A), B) Number of samples that were flagged for potential systemic and technical artefacts in sample quality control. QC=Quality control

Supplementary **Fig S2** – Overview of data before and after normalization


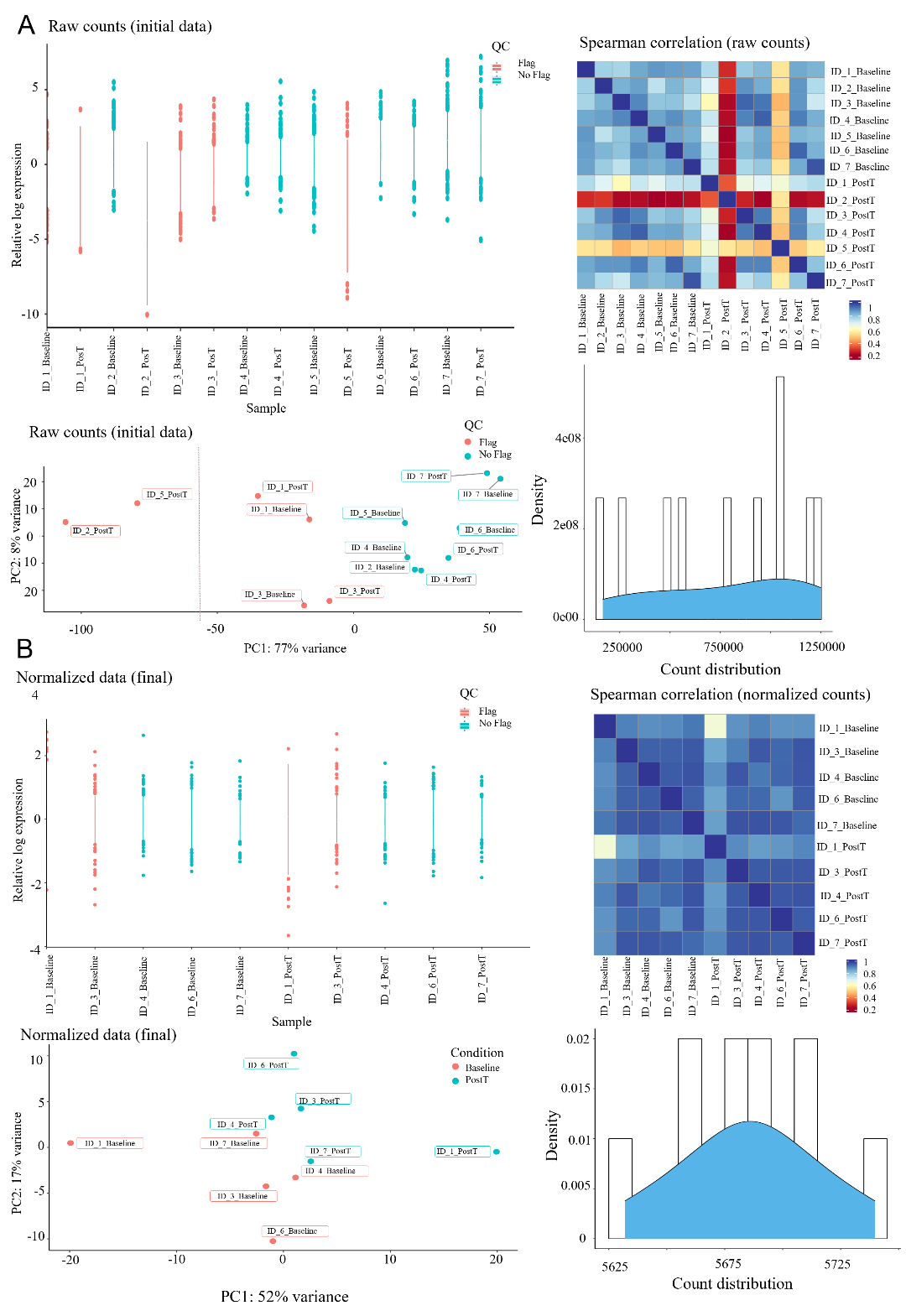


Overview of initial raw data (sample n=14) (A) and final normalized data (sample n=10) (B). Relative log expression (RLE) plots, Principal component analysis (PCA) plots, Spearman correlation heatmap, and histogram with count distribution show data characteristics before and after iterative quality control. Outliers (“ID_2_Baseline” and “ID_5_Baseline”) with their corresponding paired samples (“ID_2_PostT” and “ID_5_PostT”) were excluded in final data set. QC =Quality control, PC=Principial component, PostT=Post treatment
